# Production and characterization of ^60^Fe standards for accelerator mass spectrometry

**DOI:** 10.1371/journal.pone.0219039

**Published:** 2019-06-28

**Authors:** Dorothea Schumann, Niko Kivel, Rugard Dressler

**Affiliations:** 1 Paul Scherrer Institute Villigen, Department for Nuclear Energy and Safety, Villigen PSI, Switzerland; 2 Paul Scherrer Institute Villigen, Division Large Research Facilities, Villigen PSI, Switzerland; University of Magdeburg, GERMANY

## Abstract

Accelerator Mass Spectrometry (AMS) is one of the most sensitive analysis techniques to measure long-lived radionuclides, reaching detection limits for isotopic ratios down to 10^−15^–10^−16^ in special cases. Its application portfolio covers nearly every field of environmental research, considering processes in the atmosphere, biosphere, hydrosphere, cryosphere, lithosphere and the cosmosphere. Normally, AMS measures the content of isotopes in comparison to a validated standard. However, in some cases like for example ^60^Fe, well characterized standard materials are difficult to produce due to the extreme rareness of the isotope. We report here on the manufacturing of a set of ^60^Fe standards, obtained by processing irradiated copper from a beam dump of the high-power proton accelerator (HIPA) at the Paul Scherrer Institute (PSI). The isotopic ratios of the standards have been adjusted via a dilution series of a master solution, isotopic content of which has been characterized by Multi Collector–Inductively Coupled Plasma–Mass Spectrometry (MC-ICP-MS). In total, we produced three samples with isotopic ratios of 1.037(6)·10^−8^, 1.125(7)·10^−10^ and 1.234 (7)·10^-12^, respectively. The latter had already been applied in three pioneering AMS studies investigating the remaining signal of injected matter of nearby super novae explosions in sediment archives.

## Introduction

Recently, we observe increasing attention on the radioactive isotope ^60^Fe in nuclear astrophysics research. After first hints for a signal of a nearby supernovae (SN) explosion reported by *Knie et al*. [1,2], measuring the ^60^Fe content in meteorites and manganese crusts, four independent experimental studies, published in 2016, show strong evidence for injection of freshly produced material by a nearby super novae explosion into the Solar system in the past: 1.) *W*.*R*. *Binns et al*. observed ^60^Fe in galactic cosmic rays by use of the mass spectrometer CRIS, one of the scientific instruments installed on the Advanced Composition Explorer (ACE), launched 1997 by NASA [3], 2.) *A*. *Wallner et al*. investigated the ^60^Fe content in ocean floor samples [4], 3.) *L*. *Fimiani et al*. report on interstellar ^60^Fe on the surface of the moon [5] and 4.) *P*. *Ludwig et al*. discovered a time-resolved 2-million-year-old ^60^Fe activity in Earth’s microfossil record originating from supernova injection [6]. These findings are completed/ by transport calculations from two independent groups of astrophysical theoreticians, confirming the experimental results [7, 8]. It was for first time, that an astrophysical event in nature could be described with such high consistency by complementary branches of science, thus showing the rapidly accelerating development of interdisciplinary research in the field. The investigations help to improve the fundamental understanding of star evolution and the development of the Early Solar System in general and serve as a basis of future insight studies on the nature of the Universe.

*Wallner et al*., *Fimiani et al*. and *Ludwig et al*. used accelerator mass spectrometry (AMS) for the detection of the ^60^Fe isotopes in their samples. AMS measures isotopic ratios, in this case the number of ^60^Fe atoms against ^56^Fe. Usually, these ratios are obtained by comparison of the measured ratio to certified standard material. Due to the lack of suitable methods to produce such certified standard material in the case of ^60^Fe, for several decades the results were obtained using quasi-standards produced from heavy ion reactions [9], from irradiated copper [10] or extracted in the radioactive beam facility ISOLDE (CERN) from proton-irradiated uranium carbide [11]. None of these quasi-standards have been properly characterized by experimental methods. Their nominal values are solely based on rough approximations of the production and/or implantation rates. Uncertainties of all these materials are above 10% or even not given at all. Table 1 gives an overview on the so far used ^60^Fe quasi-standards.

**Table 1:** Summary of information on ^60^Fe quasi-standard materials.

Name	Value	Uncertainty	Production route	Ref.	Comment
no name	9·10^−10^	11%	^48^Ca(^18^O,alpha2n)^60^Fe	[9]	
KUT-CAL-B KUT-CAL-C KUT-CAL-E	7.5·10^−10^ 7.5·10^−11^ 7.5·10^−13^	15%	Proton-irradiated Cu-foil, absolute determination of the number of atoms (half-life measurement)	[10]	Factor 1.7 off according to new half-life measurements
no name	no value given	no uncertainty given	Mn-beam at CERN-ISOLDE, separation of ^60^Mn →^60^Fe	[11]	Could be ~factor 2 off (deduced from ^53^Mn measurements at TU Munich)

The need for a sufficient amount of high-quality, well-characterized standard material is clearly visible and recognized for a long time already. The difficulty consists in the availability of the isotope in such high amount, that more accurate but less sensitive measurement methods can be applied, and furthermore in the limited possibilities for accurate determination of the isotopic ratio. Since ^60^Fe is two mass units heavier than the heaviest stable iron isotope ^58^Fe, its production possibilities are limited. Double neutron capture on ^58^Fe is one production path, but this cross section (not yet experimentally determined!) is expected to be rather low. For applications requiring samples with a comparably low amount of stable iron, production via spallation reactions is the only possibility. A very unique opportunity to gain this kind of exotic isotopes is the exploitation of radioactive waste from activated components of high-power, high-energy accelerators.

PSI operates the Spallation Neutron Source SINQ, which is driven by one of the most powerful high-energetic proton accelerators world-wide (590 MeV, 2.4 mA), and is therefore best-suited as a producer of such rare radionuclides. In the frame of the ERAWAST initiative (Exotic Radionuclides from Accelerator Waste for Science and Technology) a complex program for isotope separation from different matrices has been established at PSI within the past decade [12].

A considerable amount of ^60^Fe has been identified during systematic analytical examinations in a proton irradiated copper beam dump [13]. The most isotope-rich part of the beam dump was drilled out and made available for radiochemical separation to extract ^60^Fe. A number of half-life measurements were performed with this material by several research groups, beginning in 2009 by *Rugel* et al. [14], with a new value of (2.62±0.04)·10^6^ years in remarkable disagreement with the formerly accepted value of 1.5·10^6^ years of *Kutschera* et al. [15], followed by *Wallner* et.al. (2.50±0.12)·10^6^ years [16]), confirming the "*Rugel*"-value and a third measurement in Argonne [17] with (2.69±0.28)·10^6^ years also in agreement with the latter two. Studies aimed to determine the neutron capture cross section of ^60^Fe both at thermal and stellar temperatures have been conducted as well [18, 19]. It has to be emphasized that during the past 20 years all experiments requiring ^60^Fe samples relied on material produced and manufactured at PSI.

In the following, we describe the preparation of ^60^Fe standards for AMS, comprising material with several isotopic ratios, and explain how their isotopic characterization by Multi-Collector Inductively Coupled Plasma Mass Spectrometry (MC-ICP-MS) has been performed.

## Material and methods

### Source of ^60^Fe

**Fig 1:**
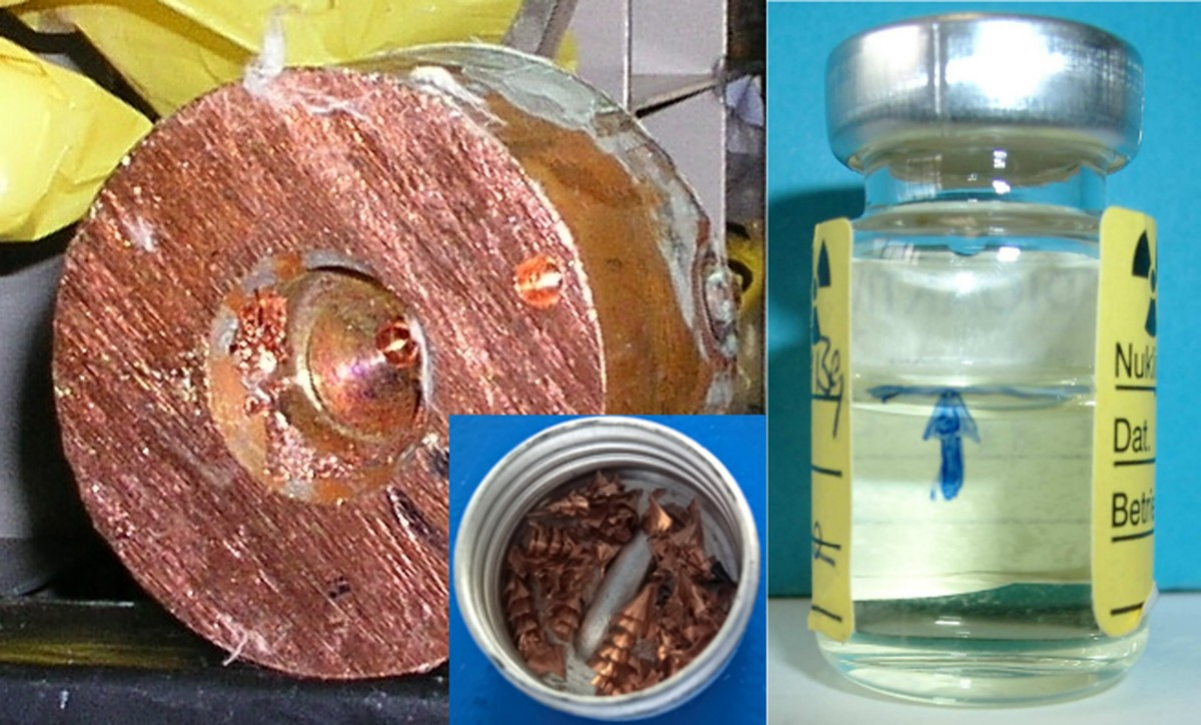
**Left: Photography of piece 2 of the copper beam dump.** The inset at the right bottom shows the chips drilled out at the central part. **Right: Photography of the master solution.** The blue arrow marked the initial level of liquid (5 ml) when used for the half-life measurement.

The primary source for the ^60^Fe standards was material drilled out of the central part of piece 2 of the copper beam dump mentioned above. Piece 2 was closest to the beam entrance, comprising the highest specific ^60^Fe activity. In Fig 1 (left), a photo of this piece is shown. The copper chips (depicted in the inset of Fig 1) were dissolved in nitric acid, 5mg of stable iron and cobalt, respectively were added, and several radiochemical procedures and purification steps were applied to isolate a sufficient amount of ^60^Fe atoms. Details of the sample preparation are described elsewhere [12]. Finally, we obtained a ^60^Fe sample dissolved in diluted hydrochloric acid, which has first been used for the half-life measurement of *Rugel* et al [14]. After finishing this experiment, we reprocessed this master solution for the manufacturing of the standard material. A photo of the master solution taken before starting the half-life measurement is depicted in Fig 1 (right).

### Determination of the isotope ratios of the master solution

The difficulty of characterizing such kind of standard material arises from the lack of measurement methods enabling to determine ultralow concentrations. Isotope ratios of 10^−8^ and lower can only be determined by AMS. However, an absolute AMS measurement is extremely challenging. Moreover, the characterization would then be correlated to the measurement method the standard is being used for. Therefore, it is necessary to start with a sufficient amount of material to fabricate a solution with high enough isotope ratio allowing for an independent measurement and perform a well characterized dilution series afterwards.

The analytical method best suited for determining the content of the iron isotopes in the master solution has proved to be MC-ICP-MS. It is a well-established and mature analytical technique which has been deployed for iron isotopic determinations since decades. As a drawback, this method cannot distinguish between isobaric isotopes. In our case, we had to consider the overlap with the stable isobar ^60^Ni, which is a) the decay product of ^60^Fe, b) is produced in the spallation reaction and c) is originally present as impurity in the copper. Normally, such interferences can be quantified by measuring the other stable isotopes of nickel and correct the number of ^60^Ni atoms in the sample using the well-known natural abundances of stable Ni isotopes. However, in this case this approach did not work initially, because due to the proton irradiation the abundances of the stable Ni isotopes are altered, and they are significantly different from the natural isotopic composition. We overcame this challenge by adding stable (natural) Ni carrier and performed repeated chemical separation of Ni by applying hydroxide precipitation. The radiochemical processes as well as the parameters of the MC-ICP-MS measurements are described in detail in [20].

The total iron amount and the isotopic composition of the master solution were determined to beN(^60^Fe) = 5.873(50)·10^15^ atoms in 4.545(8) g master solution (numbers taken from [20]). The results relevant for the standard preparation procedures are shown in Table 2.

**Table 2:** Isotopic composition of the master solution (values taken from [20]).

	^54^Fe	^56^Fe	^57^Fe	^58^Fe	^60^Fe
Isotopic abundance [%]	6.033 (19)	87.4990(50)	4.2076(68)	2.2397(70)	0.02048(12)

### Preparation of the ^60^Fe standards

Three standards were fabricated by successive dilution of the master solution with Fe_2_O_3_ (Sigma/Aldrich, 99.999%) and ultra-pure HCl (Sigma/Aldrich). All relevant quantities were determined by weighting.

#### PSI-8

0.09970 g of the master solution were mixed with 1.65277 g Fe_2_O_3_ and dissolved in concentrated HCl until complete dissolution. The total weight of the final solution amounted to 14.430667 g.

#### PSI-10

0.16146 g of the PSI-8 solution were mixed with 1.680572 g Fe_2_O_3_ and dissolved in concentrated HCl until complete dissolution. The total weight of the final solution amounted to 14.631988 g.

#### PSI-12

1.60967 g of the PSI-10 solution were mixed with 16.851904 g Fe_2_O_3_ and dissolved in concentrated HCl until complete dissolution.

After completing the dilution series, iron was precipitated with ammonia solution as Fe(OH)_3_. The hydroxide was dried and then calcinated at 400°C to obtain Fe_2_O_3_.

#### Uncertainty budget

The total uncertainty (confidence level k = 2) is determined by the uncertainty of the ICP-MS measurement (0.3%) and the corresponding error propagation in the dilution series. Uncertainties coming from the balance are negligible small (< 0.1%).

## Results

The calculation of the isotope ratios ^60^Fe/^nat^Fe yielded:

$$PSI‑8:\;I=1.037\left(6\right)\cdot 10^{‑8}$$

$$PSI‑10:\;I=1.125\left(7\right)\cdot 10^{‑10}$$

$$PSI‑12:\;I=1.234\left(7\right)\cdot 10^{‑12}$$

**Fig 2:**
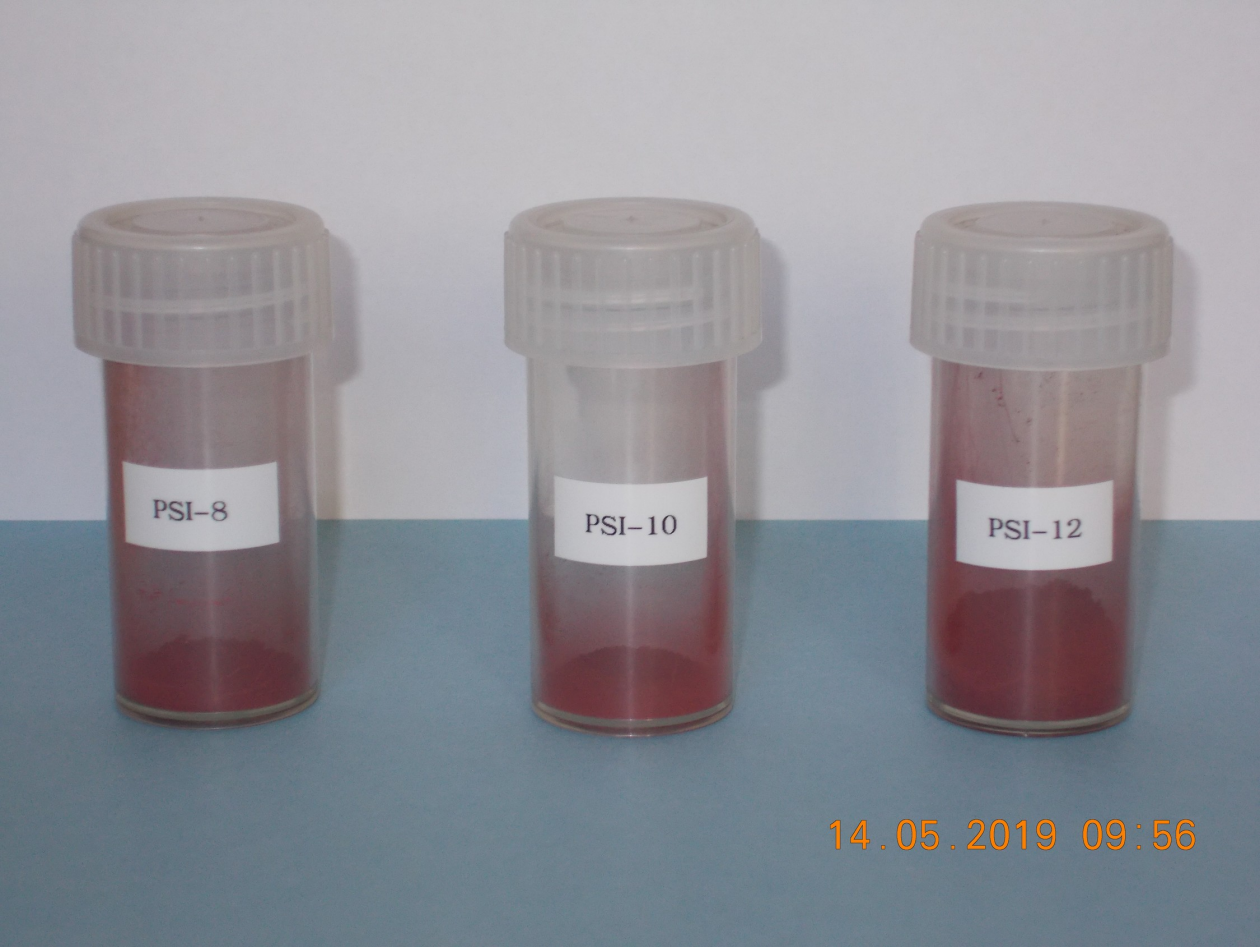
^60^Fe standard material as oxide powder (Fe_2_O_3_). Around 1.6 g of PSI-8 and PSI-10, respectively, as well as around 16 g of PSI-12 were produced.

In Fig 2, the obtained three standards in form of iron oxide powder are shown.

## Discussion

Studies on ^60^Fe are currently one of the hottest topics in understanding fundamental processes of star evolution and the development of the Early Solar System. The three recent works applying AMS [4–6] used PSI-12 as standard material. During these investigations, it turned out, that measurements using former standards showed a deviation of up to a factor two in the absolute values in comparison to the PSI ones. *Köster* mentioned a possible deviation in this order of magnitude for the standard produced at ISOLDE CERN already in 2000 [11]. With these findings, it becomes obvious, that a considerable number of former ^60^Fe data giving absolute values for ^60^Fe concentrations will have to be re-evaluated. One illustrative example is the determination of the radionuclide inventory of the PSI copper beam dump [13], containing also values for ^60^Fe. The values were obtained using former, and not characterized ^60^Fe standards. Also *Knie et al*. [2] report absolute values, which have to be corrected. Fortunately, these corrections do not influence the main outcome of the work, e.g. the detection of a SN event 2.8 Mio years ago.

We are going to make the prepared standards, which are unique world-wide, available for interested AMS groups, either on the basis of collaborations or in form of commercially purchasable goods. More batches can be produced on request. The availability of the first ever well-characterized ^60^Fe standard material will essentially improve the quality of data and enhance further high-ranking research in nuclear astrophysics. Similar AMS standards are prepared and made available in the near future for the astrophysically and geophysically interesting isotopes, e.g. ^53^Mn.
